# Did transmission of *Helicobacter pylori *from humans cause a disease outbreak in a colony of Stripe-faced Dunnarts (*Sminthopsis macroura*)?

**DOI:** 10.1186/1297-9716-42-26

**Published:** 2011-02-07

**Authors:** Alison L Every, Lynne Selwood, Natalia Castano-Rodriguez, Wei Lu, Helen M Windsor, Janet LK Wee, Agnieszka Swierczak, Barry J Marshall, Nadeem O Kaakoush, Hazel M Mitchell, Philip Sutton

**Affiliations:** 1 Centre for Animal Biotechnology, School of Veterinary Science, The University of Melbourne, Melbourne, VIC 3010, Australia; 2 Department of Zoology, The University of Melbourne, Melbourne, VIC 3010, Australia; 3 School of Biotechnology and Biomolecular Sciences, The University of New South Wales, Sydney, NSW 2052, Australia; 4 Helicobacter Research Lab, School of Biomedical, Biomolecular and Chemical Sciences, University of Western Australia, Perth, WA, Australia

## Abstract

Since the discovery that *Helicobacter pylori *causes a range of pathologies in the stomachs of infected humans, it has become apparent that *Helicobacters *are found in a diverse range of animal species where they are frequently associated with disease. In 2003 and 2004, there were two outbreaks of increased mortality associated with gastric bleeding and weight-loss in a captive colony of the Australian marsupial, the Stripe-faced Dunnart (*Sminthopsis macroura*). The presence of gastric pathology led to an investigation of potential *Helicobacter *pathogenesis in these animals. Histological examination revealed the presence of gastritis, and PCR analysis confirmed the presence of *Helicobacter *infection in the stomachs of these marsupials. Surprisingly, sequencing of 16S rRNA from these bacteria identified the species as *H. pylori *and PCR confirmed the strain to be positive for the important pathogenesis factor, *cagA*. We therefore describe, for the first time, an apparent reverse zoonotic infection of Stripe-faced Dunnarts with *H. pylori*. Already prone to pathological effects of stress (as experienced during breeding season), concomitant *H. pylori *infection appears to be a possible essential but not sufficient co-factor in prototypic gastric bleeding and weight loss in these marsupials. The Stripe-faced Dunnart could represent a new model for investigating *Helicobacter*-driven gastric pathology. Infections from their human handlers, specifically of *H. pylori*, may be a potential risk to captive colonies of marsupials.

## Introduction

The discovery of *Helicobacter pylori *eventually led to a realization that infection of the human stomach with this bacterium is a key etiological factor in the development of peptic ulcer disease and gastric adenocarcinoma [1-3]. Another important consequence of that discovery has been the subsequent identification of an ever growing, seemingly ubiquitous family of *Helicobacter *species that infect the gastrointestinal tract of virtually all animals examined. In fact, many *Helicobacter *infections are believed to be pathogenic and possibly responsible for a wide range of conditions in numerous animal species (reviewed in [4,5]). For example, natural *Helicobacter *infections have been linked with gastritis in small and large cats, dogs, pigs and ferrets [6-10], abortion in sheep [11], colitis and hepatitis in rhesus monkeys [12], ulcers in dolphins and whales [13] and diarrhea in cats and parrots [14,15].

In this report we describe two outbreaks of deaths in a captive colony of an Australian marsupial, the Stripe-faced Dunnart (*Sminthopsis macroura*), that were associated with bleeding in the stomach possibly due to infection by the human pathogen, *H. pylori*.

## Materials and methods

### Stripe-faced Dunnarts

The Stripe-faced dunnarts were housed at the Department of Zoology, University of Melbourne. The colony was held within cages maintained within a purpose built temperature regulated building, in a physical environment in conditions typical for those for housing mice, except that natural lighting was maintained. Animal housing in specially designed cages, the diet formulated for carnivorous insectivorous marsupials and reproductive monitoring followed the outline given previously [16]. No other animal species were housed in this building. The dunnarts were provided water ad libitum. No dunnarts were killed specifically for this study; stomachs were only collected from animals euthanized either due to illness or for other experimentation approved by the University of Melbourne Science, Optometry & Vision Sciences and Land & Environment animal ethics-committee. Animals were weighed at weekly intervals during routine animal husbandry.

### Rapid urease test (CLO test)

Stomach biopsies of 1-2 mm were placed in the gel of a rapid urease test (CLOtest; Kimberly-Clark, Roswell, Georgia, USA). Tests were monitored over a 24 h period for a color change from yellow to red which indicated the presence of a urease-producing organism in the stomach.

### Histological assessment of pathology

Stomach halves were fixed in 10% neutral buffered formalin, embedded in paraffin, then 4 μm-thick sections cut and stained with hematoxylin and eosin.

### Detection of *Helicobacter *infection by genus-specific PCR

For assessment of *Helicobacter *infection by PCR, feces were collected, or Dunnart stomachs were opened along the inner curvature and the whole organ homogenized (GmbH Polytron homogenizer; Kinematica, Switzerland). Mucosal scrapings were also collected from the intestine (large and small). DNA was extracted from feces and intestinal scrapings using a QIAamp stool DNA kit (Qiagen, Hilden, Germany), and from the homogenized stomach using the QIAamp tissue DNA kit (Qiagen) according to manufacturer's instructions. A *Helicobacter *genus PCR targeting a 374 bp fragment of the 16S rRNA gene was performed as previously described [17].

### Approaches to cultivate Dunnart gastric *Helicobacter*

Dunnart stomachs were collected and homogenized in Brain Heart Infusion broth (BHI; Oxoid, Basingstoke, UK). Homogenate was added to horse blood agar or GSSA plates (as described previously [18]) which were incubated in an anaerobic jar with a microaerophilic gas-generating kit (Oxoid, Basingstoke, UK) for up to 10 days at 37°C. For the filtration technique, homogenate was placed onto 0.65 μm Whatman filter paper (GE Healthcare, Rydalmere, Australia), placed on HBA plates, and incubated at 37°C under microaerophilic conditions for 2 h. Filters were then removed and plates incubated at 37°C under microaerophilic conditions for up to 10 days.

### Gene sequencing of *16S ribosomal RNA*, *cagA *and *ureA*

Nine DNA samples extracted from Dunnart stomach and intestinal scrapings were subjected to sequencing. PCRs for *Helicobacter *genes were performed as previously described for *16S rRNA *[17], *cagA *[19] and *ureA *[20]. DNA sequencing of the positive PCR products was done by means of the BigDye terminator chemistry (Applied Biosystems; Foster City, USA). Sequencing of both the 5' and 3' end of the amplicons occurred in a volume of 20 μL consisting of 3.5 μL sequencing buffer, 1 μL BigDye v3.1, 10 pmol/μL of the required primer, 40-100 ng DNA, and water to make up the final volume. The sequencing program consisted of 96°C for 1 min, 30 cycles of 96°C for 10 seconds, 50°C for 5 min and 60°C for 4 min. The sequences were compared to gene sequences of known identities using the BLASTn search program available through the National Centre for Biotechnology Information (NCBI) website [21].

## Results

### Deaths in a Stripe-faced Dunnart colony - 2003

A colony of Stripe-faced Dunnarts has been in existence for 26 years, initially at La Trobe University (Melbourne, Australia), and then at the Department of Zoology, University of Melbourne. The colony normally has between 60-100 animals in which the sex ratio is 1/1. Death from illness is rare in these animals and in a normal year, total deaths occur at a rate of about 10% or less of the number of animals in the colony. Most deaths occur as a result of injuries obtained during mating (the breeding season runs from July to December) or old age.

**Figure 1:**
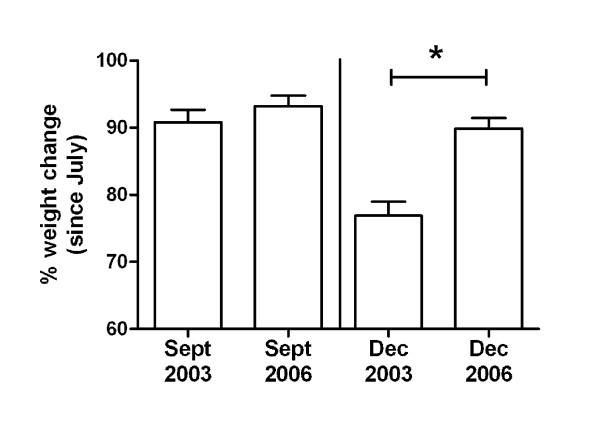
**Increased loss in body weight in Stripe-faced Dunnart during disease outbreak**. Values shown present the mean change in body weights ± S.E.M. of Stripe-faced Dunnarts during July-December (calculated as the weight of individual animals in September and December as a percentage of their weight in July). Data shown is from one year of the disease outbreak (2003; *n *= 25) and a normal year when no increased mortality was observed (2006; *n *= 35). Both males and females are included and the data combined, as both sexes lost similar body weights. There was no significant difference in body weights in September 2003 and 2006. However, the animals lost significantly more weight in the period up to December 2003, compared to the same period in 2006 (* *p *< 0.001; ANOVA).

In 2003 however, there was a dramatic rise in the mortality rate of these animals, with 29% of the Dunnarts (30/104; similar numbers of males and females) dying in the 6 month period from July to December. The dying animals were 1-2 years old and the deaths were not related to old age. Dunnarts usually lose body weight during the breeding season and this weight loss was also notably increased during 2003. Male Dunnarts typically lose an average 12% body weight from July to December, but in 2003 they lost an average 27.7%. Similarly, female Dunnarts lost an average 30.3% during the same period in 2003, compared with a typical average loss of 16%. Figure 1 shows the relative weight loss observed by these Dunnarts in 2003, as compared to weight loss observed in 2006, a typical year in which there was no increased mortality.

The only evident pathology observed at post mortem was that almost half of the animals that died or were culled due to being very sick had severe gastric hemorrhage. Apart from increased weight loss, most animals seemed healthy until 1-2 days before death, although a few had black stools (consistent with gastrointestinal bleeding) and signs of anemia (pale snouts and gums).

### Deaths in a Stripe-faced Dunnart colony - 2004

**Figure 2:**
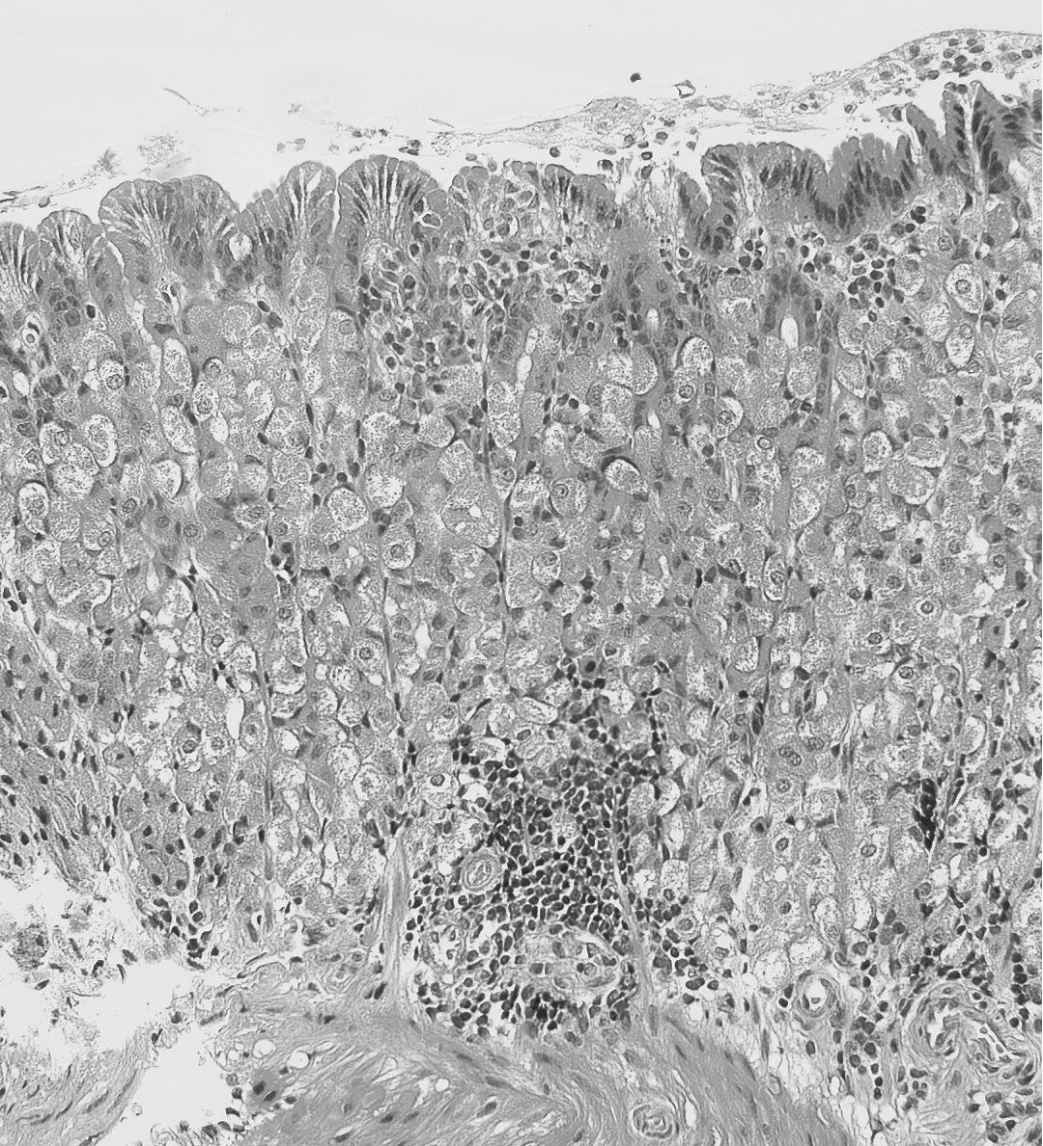
**Inflammation in the gastric lamina propria of a *Helicobacter*-infected Dunnart**. An image is shown from an H&E stained section of gastric corpus, from a *Helicobacter*-infected Dunnart (magnification ×100). The stomach was collected in May 2004.

The gastric bleeding present in these animals raised the possibility of gastric ulceration as a result of *Helicobacter *infection. Therefore, in May 2004, stomachs from two asymptomatic female Dunnarts were tested for *Helicobacter *infection using the CLO test. These were found to be positive, supportive of a gastric *Helicobacter *infection. Histological analysis of these stomachs revealed the presence of a relatively mild inflammatory process, marked by lymphoid aggregates in the lamina propria (Figure 2), although no colonizing bacteria in either spiral or coccoid form could be observed in these sections. No severe pathology was evident in these particular sections, although these stomachs were collected outside the breeding season when increased mortality and gastric bleeding was not occurring.

Between July and December 2004 a second incident of increased mortality occurred, with 24 out of 81 (30%) of the Dunnart colony dying, 6 of which had gastric bleeding.

In several cases in 2003 and 2004, females that were deemed to be pregnant from reproductive monitoring had aborted the litter when they were examined to obtain embryos.

### *Helicobacter *infection in the Dunnart colony 2005-2008

In 2005, and in subsequent years, the mortality rate returned to normal levels (~10% per year) and no further cases of gastric bleeding were observed. However sporadic attempts were made to further examine the apparent gastric *Helicobacter *infection in these animals. These attempts were limited by the availability of animals for such studies.

In 2006, the presence of *Helicobacter *in fecal samples collected from male (*n *= 20) and female (*n *= 20) Dunnarts were assessed by genus specific PCR. Using this approach, the feces of equivalent numbers of male (7/20) and female (7/20) Stripe-faced Dunnarts were shown to be colonized with *Helicobacter *species, providing evidence of the presence of *Helicobacters *in the GI tract of at least some animals in this colony. Attempts to cultivate *Helicobacter *from these samples were unsuccessful.

In July 2007, stomachs and intestinal scrapings collected from Dunnarts were analyzed by genus-specific PCR. These showed that 10/12 of the animals examined had gastric *Helicobacter *infection. Again, attempts to cultivate *Helicobacter *from the gastric samples were unsuccessful.

### Species identification of *Helicobacter *infection in the Dunnart stomach

To identify whether a known or novel *Helicobacter *species was infecting the Stripe-faced Dunnart colony, the *16S ribosomal RNA *(rRNA) *Helicobacter *gene from these gastric samples was partially sequenced. This indicated that the *16S rRNA *gene sequence of *Helicobacter *infecting the stomachs of Stripe-faced Dunnarts corresponded to *H. pylori *strains originally isolated from different regions of the human gastrointestinal tract including the stomach and intestine. The identity percentages obtained with the sequences were 100% (Table 1).

**Table 1:** BLASTn retrieval of ***Helicobacter pylori ***gene sequences

BLASTn Retrieve	GenBank accession number	Score (bits)	Identity	E-values
*Helicobacter pylori *clone P13 *16S ribosomal RNA *gene	EF684928.1	699	100%	0.0

*Helicobacter pylori *strain CD2 *16S ribosomal RNA *gene	HM243132.1	699	100%	0.0

*Helicobacter pylori *strain HP504 *16S ribosomal RNA *gene	GU449115.1	695	100%	0.0

*Helicobacter pylori *v225d *16S ribosomal RNA *gene	CP001582.1	695	100%	0.0

*Helicobacter pylori *strain 407D5 *16S ribosomal RNA *gene	HM099656.1	695	100%	0.0

*Helicobacter pylori *strain 15818 cytotoxin associated protein III (*cagA*) gene	AF083352.1	1288	96%	0.0

*Helicobacter pylori *NCTC 11639 CagA (*cagA*) gene	GQ161099 .1	1282	96%	0.0

*Helicobacter pylori *strain 114C UreA (ureA) gene	GQ403154.1	630	96-98%	3e^-164 ^1e^-177^

*Helicobacter *genus-specific PCR products derived from Dunnart gastric DNA extracts were compared with the *H. pylori *Genbank sequences listed in the Table. The Dunnart samples exhibited 100% sequence identity with 16s rRNA from five human clinical isolates of *H. pylori*, and 96-98% sequence identity with cagA and *ureA *from two human clinical isolates of *H. pylori*.

The important *H. pylori *virulence factor, *cag*PAI (*cag *pathogenicity island) encodes a type-IV secretion system involving cagA. Not all *H. pylori *strains possess this virulence factor, and individuals infected with *cag*PAI positive strains typically develop more severe gastritis, and have a greatly increased chance of progressing to disease. Urease, an enzyme composed of two subunits *ureA *and *ureB*, is essential for the pathogenesis of *H. pylori *in the human stomach because of its ability to neutralize gastric acid through the production of bicarbonate. Analysis using specific PCR primers revealed that the infecting *Helicobacter *were *cagA *and *ureA *positive, and gene sequencing revealed 96% identity with *cagA *and 96-98% identity with *ureA *from *H. pylori *(Table 1).

### Natural loss of *H. pylori *infection in the Stripe-faced Dunnart colony

When samples were collected from 18 female Dunnarts in July of 2008 surprisingly, all of these animals were *Helicobacter *negative indicating the colony had lost the infection. There is no obvious explanation for the loss of *H. pylori *infection in these animals, as there was no change in husbandry, nor were the Dunnarts treated with any antibiotics during this period.

For clarity, a chronology of the above events is summarized in Table 2. The Dunnart colony in the Zoology Department at the University of Melbourne was closed in 2009, precluding further study.

**Table 2:** Chronology of events

Date	Event
July-Dec 2003	Increased mortality observed in Dunnart colony with gastric bleeding.

May 2004	Two Dunnart stomachs were tested and shown to be *Helicobacter *positive by CLO test. Histology revealed mild gastritis.

July-Dec 2004	Increased mortality observed in Dunnart colony with gastric bleeding.

2006	Dunnart feces shown to be *Helicobacter *positive by PCR.

2007	Dunnart stomachs shown to be *Helicobacter *positive by PCR. The infection was shown to be *H. pylori *by partial sequencing of *16S rRNA*, *cagA *and *ureA*.

2008	Dunnart colony shown to be *Helicobacter *negative by PCR of gastric tissues.

## Discussion

Two outbreaks of increased mortality in a colony of the Australian marsupial, the Stripe-faced Dunnart in 2003 and 2004 were associated with gastric bleeding and occurred coincidental with a transient infection of the stomachs of animals in this colony with the human pathogen, *H. pylori*. Notably, this infecting strain possessed the virulence factor *cagA*, which suggests the presence of the *cag*PAI type-IV secretion system, associated with an increased risk of developing gastric cancer or peptic ulcers in humans. While only five of the nine Dunnart samples analyzed were *cagA *and *urea *positive, this may be explained by the low amount of *Helicobacter *DNA in these samples, although the possibility of heterogeneity in the infecting strains cannot be excluded.

It was unfortunate we were unable to cultivate *Helicobacter *from the Dunnart stomachs, as this may have allowed us to study the level of heterogeneity, if any, of these bacteria in the colony. The initial attempts to cultivate *Helicobacter *were performed in Perth, Western Australia, on samples sent from Melbourne. The delay caused by collection and transfer of these samples may have limited our ability to isolate the infecting bacteria. However, in later studies we did attempt to cultivate them from fresh gastric tissue. Attempts on fresh tissue using HBA plates failed, not too surprisingly, due to fungal overgrowth. When selective GSSA plates were used, we did manage to initially culture a motile bacterium, but this was subsequently overgrown by a coccoid organism. We next attempted a filtration approach, which successfully restricted contamination by the coccoid organism, but unfortunately also did not allow us to isolate *Helicobacter*. This may suggest the *Helicobacter *present in the Dunnarts either had poor motility (an important feature required for migration of bacteria through the filter paper) or were only present in low levels.

As *H. pylori *infection does not appear to be part of the normal flora of these animals (as it spontaneously cleared between 2007 and 2008) we theorize that *H. pylori *may have entered this colony as an infection from their human handlers. The concomitant presence of stomach infection with *H. pylori *at the time of the gastric bleeding incidents strongly suggests this bacterium may have contributed to the disease outbreak. Clearly, *H. pylori *infection alone was insufficient to cause the outbreaks as the infection was present during 2005-2007 without any increase in mortality. However, it may well have been an important co-factor in this process.

The loss of *H. pylori *infection between 2007 and 2008 is intriguing, with the reason for this occurrence remaining unknown. Given *Helicobacter *were present for at least three years, it was not a short-term phenomenon but was present over many breeding cycles. It therefore seems unlikely that loss of this infection was due to the *Helicobacter *being out-competed by native flora, although this possibility cannot be excluded. It is also possible that the animals were not transmitting the infection between themselves, but perhaps were being constantly infected by a human handler. If this were the case, the loss of infection may have been due to a change in handler between 2007 and 2008 breaking the infection transmission.

Stress appears to have been an important co-factor in the disease etiology, as the outbreaks occurred during the breeding season in these animals. The breeding season is extremely stressful for both male and female Dunnarts, and in an average year, is accompanied by a considerable loss in body weight. Marsupials can be remarkably sensitive to stress, and it has been shown that stress events can have a major affect on their immune system [22].

Despite this, there must be another co- or causative factor involved in the 2003-2004 outbreaks, as the Dunnarts bred while infected with *H. pylori *in 2005-2007 without any increase in death rate. This co-factor remains unidentified, but does not appear to be anything related to husbandry as there were no changes in the housing conditions of the colony that were associated with either the disease outbreaks or loss of *H. pylori *infection.

In conclusion, we propose that infection with the human pathogen *H. pylori *may have been an essential, though not sufficient co-factor in an outbreak of gastric bleeding resulting in death in a colony of Stripe-faced Dunnarts. The apparent accidental infection of these marsupials by their human handlers, and the association with coincidental gastric pathology, suggests that these animals may provide an interesting novel animal model for the study of *H. pylori *pathogenesis. Further, this observation suggests a potential risk factor to captive marsupials to infections carried by their human handlers.

## Competing interests

The authors declare that they have no competing interests.

## Authors' contributions

LS made the initial observation, ran the Dunnart colony and provided samples for analysis. AE, WL, HW, JW, AS and BM performed *Helicobacter *PCR analyses. NCR, NK and HM performed gene sequencing analyses. AE and PS wrote the manuscript. PS coordinated the study. All authors read and approved the final manuscript.
